# Arterial specification precedes microvascular restitution in the peri-infarct cortex that is driven by small microvessels

**DOI:** 10.1177/0271678X241270407

**Published:** 2024-08-07

**Authors:** Nina Hagemann, Yachao Qi, Ayan Mohamud Yusuf, AnRan Li, Xiaoni Zhang, Philippa Spangenberg, Anthony Squire, Thorsten R Doeppner, Fengyan Jin, Shuo Zhao, Jianxu Chen, Axel Mosig, Matthias Gunzer, Dirk M Hermann

**Affiliations:** 1 Department of Neurology, 39081 University Hospital Essen, University of Duisburg-Essen, Essen, Germany; 2 Center for Translational Neuro- and Behavioral Sciences, University Hospital Essen, Essen, Germany; 3 Institute for Experimental Immunology and Imaging, 39081 University Hospital Essen, University of Duisburg-Essen, Essen, Germany; 4 Imaging Center Essen, University Hospital Essen, University of Duisburg-Essen, Essen, Germany; 5 Department of Hematology, First Hospital of Jilin University, Changchun, Jilin, China; 6 Leibniz-Institut für Analytische Wissenschaften (ISAS), Dortmund, Germany; 7 Bioinformatics Group, Center for Protein Diagnostics, Ruhr-University, Bochum, Germany

**Keywords:** Angiogenesis, FTY720, light-sheet microscopy, microvascular network analysis, middle cerebral artery occlusion

## Abstract

Evaluation of microvascular networks was impeded until recently by the need of histological tissue sectioning, which precluded 3D analyses. Using light-sheet microscopy, we investigated microvascular network characteristics in the peri-infarct cortex of mice 3–56 days after transient middle cerebral artery occlusion. In animal subgroups, the sphingosine-1-phosphate analog FTY720 (Fingolimod) was administered starting 24 hours post-ischemia. Light-sheet microscopy revealed a striking pattern of microvascular changes in the peri-infarct cortex, that is, a loss of microvessels, which was most prominent after 7 days and followed by the reappearance of microvessels over 56 days which revealed an increased branching point density and shortened branches. Using a novel AI-based image analysis algorithm we found that the length density of microvessels expressing the arterial specification marker α-smooth muscle actin markedly increased in the peri-infarct cortex already at 7 days post-ischemia. The length and branch density of small microvessels, but not of intermediate or large microvessels increased above pre-ischemic levels within 14–56 days. FTY720 increased the length and branch density of small microvessels. This study demonstrates long-term alterations of microvascular architecture post-ischemia indicative of increased collateralization most notably of small microvessels. Light-sheet microscopy will greatly advance the assessment of microvascular responses to restorative stroke therapies.

## Introduction

In the ischemic brain, the evolving infarct is surrounded by peri-infarct tissue, in which cerebral blood flow is comprised but does not reach critical thresholds for energy breakdown.^1,2^ In this peri-infarct tissue, disseminate neuronal injury evolves over several days.^2,3^ Cerebral microvessels degenerate.^3,4^ This delayed injury is followed by a phase of microvascular remodeling and angiogenesis,^4,5^ which enables brain parenchymal tissue plasticity.^6^ In the peri-infarct tissue, a close and direct association between vascular remodeling, the restoration of blood flow and functional neurological recovery has been demonstrated.^7^ Hence, the analysis of vascular remodeling is crucial for the understanding of brain repair processes, and it may also help in the identification of new treatments that promote stroke recovery.

The evaluation of microvascular remodeling post-stroke is impeded by the need of brain tissue sectioning in conventional microscopy. The thickness of brain sections is typically in the range of 10–25 µm, which allows examining short vessel profiles only. By meticulous reconstruction of high quality brain sections, 3D image stacks can be obtained, which enable microvascular network analyses.^8^ Such reconstructions have already been used for demonstrating the reappearance of blood vessels in the peri-infarct cortex following middle cerebral artery occlusion (MCAO).^8^ Advancements in microscopic imaging, such as 2-photon microscopy and light-sheet fluorescence microscopy (LSFM), have recently improved the visualization and analysis of brain microvessels.^9,10^ 2-photon microscopy revealed post-ischemic alterations in vessel morphology, such as vessel narrowing, tortuosity and loss, and blood flow in large and small vessels.^9,11^ To date, comprehensive investigations of vascular remodeling days or weeks post-stroke have not been performed using this technology.

Here, we used LSFM in combination with the recently developed VesselExpress pipeline, which allows fully automated segmentation, skeletonization and graph analysis of microvascular networks,^12^ to examine microvascular remodeling in the peri-infarct cortex of mice exposed to two durations of transient MCAO. Microvascular network characteristics were assessed for different vessel calibers at various time-points from 3 to 56 days post-MCAO. Using a recently published immunolabeling protocol,^13^ we quantified vascular networks with arterial or venous specification. This was made possible by a novel artificial intelligence (AI)-based VesselExpress algorithm, which allows detecting hollow vessel structures with high sensitivity and robustness. Endothelial sphingosine-1-phosphate (S1P) receptors play a central role in regulating microvascular responses in the ischemic brain.^14,15^ To study the effects of an angiogenic treatment, we investigated how the S1P analog FTY720 (Fingolimod), which is the first orally administered drug for the treatment of relapsing-remitting multiple sclerosis,^15^ influences microvascular network remodeling in the peri-infarct cortex. FTY720 has been shown to improve neurological recovery in stroke patients in prospective randomized studies.^16,17^ For this reason, FTY720 is considered as a promising candidate for larger multicentric stroke trials.

## Methods

### Animals, animal maintenance and treatment

Animal experiments were performed in accordance with the regulations of the National Institute of Health Guidelines for the Care and Use of Laboratory Animals in compliance with ARRIVE guidelines and the permission of Landesamt fuer Natur, Umwelt und Verbraucherschutz (LANUV) which is part of the Ministry for Environment, Agriculture, Conservation and Consumer Protection (MULNV) of the State of North Rhine-Westphalia. Male C57Bl/6j mice (25–30 g body weight, 10–12 weeks; Envigo, Horst, Netherlands) were kept in a 12 h–12 h light/dark cycle with free access to food and water in groups of 5 animals per cage. Subgroups of mice were intraperitoneally treated with vehicle or FTY720 (1 mg/kg; Fingolimod, Sigma-Aldrich, Deisenhofen, Germany) once per day starting at 24 hours after MCAO. A priori analysis was performed assuming an effect size of 0.4–0.6 (depending in the parameter evaluated) with an alpha error of 0.05 and a power of 80%. Animals were randomly assigned to experimental groups. Experimenters were blinded according to group allocation and during image analysis. In total 325 mice were enrolled. As exclusion criteria, mice were removed from the study when suffering from respiratory abnormalities or from severe nurturing handicaps resulting in a weight loss >20%. Detailed information regarding experimental groups and animal exclusion is shown in Supplemental Table 1.

### Focal cerebral ischemia

**Figure 1.**
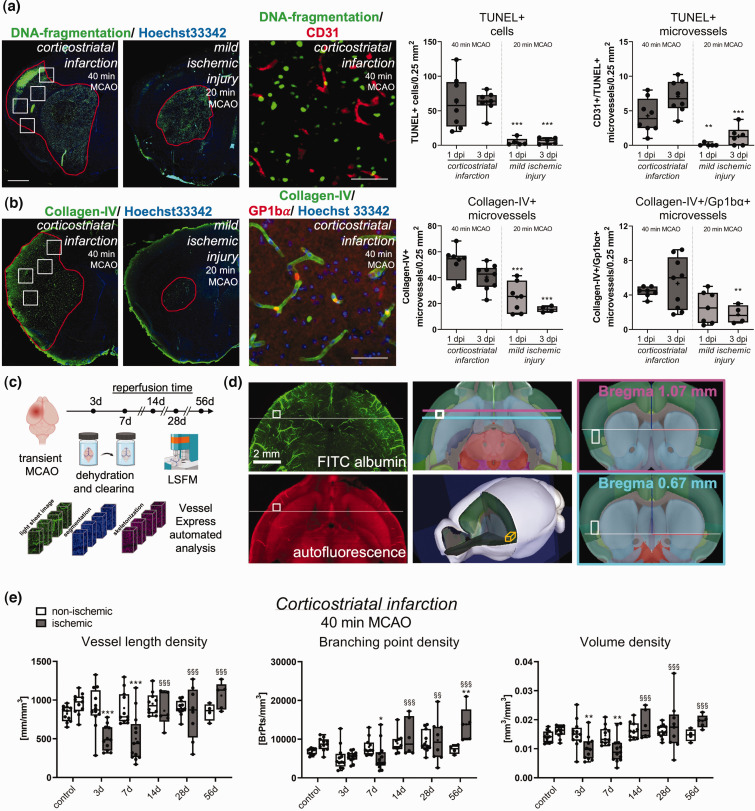
Ischemic injury characteristics and microvascular remodeling in the peri-infarct cortex. (a) Analysis of TUNEL+ cells and TUNEL+ microvessels and (b) collagen-IV+ microvessels and collagen-IV+ microvessels exhibiting Gp1bα+ platelet aggregates in four ROIs (indicated by white rectangles) of the previously ischemic S2 somatosensory cortex of 40 min and 20 min MCAO mice at 1 and 3 days post-ischemia (dpi). Red lines in the images on the left outline areas exhibiting DNA fragmentation (in a) and collagen-IV staining (in b), respectively. While 40 min MCAO induced corticostriatal infarcts, 20 min MCAO lesions were largely confined to the striatum. The tissue area exhibiting collagen-IV labeling in 40 min MCAO mice, which was devoid of major cell injury, was chosen for LSFM analysis. Images on the right depict the spatial relationship of DNA-fragmented cells to CD31+ endothelial cells (in a) and the presence of Gp1bα+ platelet aggregates in collagen-IV+ microvessels (in b). (c) Experimental overview showing time-points of vascular analysis. Brains were dehydrated and cleared. Images were segmented, skeletonized and analyzed by VesselExpress. (d) Left: Representative LSFM images in transverse (scanning) direction showing FITC-albumin-stained vessels (top) or 561 nm autofluorescence (bottom). Center: Allen Mouse Brain Atlas images representing transverse view (top) or whole-brain view (bottom) displaying location of the ROI used for peri-infarct vascular analysis. Right: Coronal view displaying the ROI at its starting point and endpoint. (e) Microvascular network analysis in the previously ischemic peri-infarct cortex and contralateral non-ischemic cortex of mice exposed to 40 min MCAO. Microvascular length density, branching point density and volume density were assessed by FITC-albumin hydrogel. Note the reduction of vessel length density, branching point density and volume density at 3 and 7 dpi, which was followed by the restitution of length density and volume density at 14–56 dpi, while branching point density increased ∼2 times above pre-ischemic levels. Data are box plots with medians (lines)/means (plus symbols) ± IQRs with minimum and maximum data as whiskers. Data of individual animals are shown as dots. *p < 0.05/**p < 0.01/***p < 0.001 compared with contralateral non-ischemic; ^§§^p < 0.01/^§§§^p < 0.001 compared with 7 dpi (i.e., nadir of vessel network). Sample size: n = 11 (non-ischemic control), n = 12 (3 dpi), n = 13 (7 dpi), n = 10 (14 dpi), n = 11 (28 dpi) and n = 5 (56 dpi) animals, respectively. Scale bars: 1 mm (in a and b, left/center); 50 µm (in a and b, right); 2 mm (in d).

MCAO was induced using an intraluminal filament technique as previously reported.^18,19^ Briefly, male C57Bl/6j mice were anesthetized using 1% isoflurane (30% O_2_ and remainder N_2_O), while body temperature was maintained between 36.5 and 37.0°C using a feedback-controlled heating system (Fluovac, Harvard Apparatus, Holliston, MA, USA). An incision at the neck midline was made to dissect the left common and external carotid arteries. The common carotid artery was sutured and the internal carotid artery was transiently clipped. To occlude the MCA, a silicon resin-coated nylon monofilament was inserted through a small incision of the left common carotid artery and advanced to the left internal carotid artery up to the offspring of the left MCA from the circle of Willis. Reperfusion was established by the withdrawal of the monofilament. In two sets of experiments, proximal MCAO was induced for 40 or 20 min, resulting in combined corticostriatal infarcts and disseminate cell injury restricted to the striatum, respectively (Figure 1(a)). In all animals, laser Doppler flow was monitored during ischemia and up to 20 min after reperfusion using a flexible 0.5 mm fiber-optic probe (Perimed, Rommerskirchen, Germany) attached to the intact skull overlying the MCA territory (2 mm posterior, 6 mm lateral from bregma). After the surgery, wounds were sealed, anesthesia was discontinued and animals were placed in a warming cabinet (37.0°C) for 1 h to recover. Analgesia was ensured by subcutaneous injection of 0.1 mg/kg buprenorphine (Temgesic; Essex Pharma, Munich, Germany) before surgery and subcutaneous injection of 4 mg/kg carprofen (Bayer Vital, Leverkusen, Germany) directly after MCAO and thereafter daily for 3 days at 24-h-intervals.

### Hydrogel preparation

For labeling of microvessels, hydrogel containing FITC-conjugated albumin was prepared as described previously.^10^ Briefly, a solution of 2% (w/v) gelatin (Sigma-Aldrich, Deisenhofen, Germany) was dissolved in 0.1 M phosphate buffered saline (PBS) (Merck-Millipore, Darmstadt, Germany) at 60°C and allowed to cool down to 40°C with constant stirring. Then, FITC-conjugated albumin (Sigma-Aldrich) was added to the gelatin solution at a concentration of 0.1% (w/v). The gel was filtered using filter paper (GE Whatman, Dassel, Germany) and continuously stirred at 30°C to avoid excessive evaporation.

### Animal sacrifice and hydrogel perfusion

Mice were deeply anesthetized in isoflurane and transcardially perfused with 40 mL of PBS containing 50 U/mL heparin (Ratiopharm, Ulm, Germany), followed by perfusion of 40 mL of 4% paraformaldehyde (PFA) (Merck-Millipore) in PBS. Thereafter, 10 mL of FITC-albumin hydrogel was transcardially perfused in animals used for light sheet microscopy, and the mouse bodies were then placed with the head down into ice water over 15 min. The brains of all mice were carefully removed and incubated in 4% PFA in PBS at 4°C overnight. In a subset of mice, platelets were labeled by intravenous injection of 5 µg anti-GP1bβ antibody (X649, emfret, Eibelstadt, Germany) 30 minutes before FITC-albumin hydrogel perfusion.

### Whole brain clearing

For clearing adult mouse brains perfused with hydrogel, we used established protocols using a modified three-dimensional imaging of solvent-cleared organs (3DISCO) technology as previously reported.^10^ Incubation of brains in tetrahydrofuran (THF; Sigma–Aldrich) was performed for 12 h each in increasing concentrations (30%, 60%, 80%, and 100%) at room temperature with constant agitation at 300 rpm using a horizontal shaker under a laminar flow hood. To ensure complete dehydration, samples were immersed in solutions of the last THF gradient (100%) twice. Then, samples were incubated in ethyl cinnamate (ECi; Sigma-Aldrich) for 12 h with continued agitation and stored in this solvent until image acquisition. All incubation steps were done in 30 mL of each solvent in dark brown glass bottles.

### Whole brain staining

In subgroups of mice, whole brains were stained for arterial^20^ and venous^13^ specification markers using a recently published immunolabeling-enabled 3D imaging (iDISCO) protocol.^13^ Briefly, PFA perfused brains were removed from MCAO mice at 7 or 28 days post-ischemia (dpi) and stored for 2 h in PFA before dehydration was performed using increasing concentrations of methanol (starting from 20% followed by 40%, 60%, 80% and 100% for 1 h each). After an additional incubation in 100% methanol for 2 h, brains were incubated in 66% dichloromethanol overnight and subsequently immersed in 100% methanol twice for 4 h each. After this, a bleaching step was included using 5% H_2_O_2_ in methanol overnight at 4°C. Brains were then rehydrated in 60%, 40% and 20% methanol for 1 h each followed by two washing steps in PBS for 15 min each. For permeabilization, brains were first incubated for 1 h in PBS containing 0.2% Triton X-100 followed by incubation in PBS containing 0.2% Triton X-100, 20% DMSO and 2.3% glycine for 24 h. For immunostaining, brains were incubated in blocking buffer (PBS containing 0.2% Triton X-100 and 0.2% gelatin) for 24 h at 37°C followed by a 10-day incubation in goat anti-α-smooth muscle actin (SMAα; 1:1000; NB300-978; Novus, Wiesbaden, Germany) and rabbit anti-von Willebrand factor (vWF; 1:800; A0082; Agilent, Santa Clara, CA, U.S.A.) antibody at 37°C. Brains were washed 3 times in PBS containing 0.2% Triton X-100 before a 10-day incubation in Alexa Fluor 555-labeled donkey anti-rabbit IgG and Alexa Fluor 647-labeled donkey anti-goat IgG (1:500 each; A-31572 and A-21447; Invitrogen, Waltham, MA, U.S.A.) at 37°C. Brains were washed as before and dehydrated using increasing methanol concentrations as described above for 1 h each. After an incubation with 100% methanol overnight, brains were incubated in 66% dichloromethanol in methanol for 3 h followed by two incubations in 100% dichloromethanol for 15 min each. Clearing of brains was performed using ECi as described above.

### Light sheet data acquisition and image processing

For reproducible and open research, we strictly followed the new community-developed checklist for reporting bioimage analysis workflows.^21^ The details for all minimal and recommended requirements are provided. Cleared whole mouse brains were imaged using an Ultramicroscope-2 (LaVision BioTec, Bielefeld, Germany) light sheet microscope. Ultramicroscope-2 is based on an Olympus MVX10 zoom microscope, which, when combined with a 2 × 0.5 numerical aperture objective, provided a magnification range from 1.26 to 12.6. Ultramicroscope-2 was equipped with bidirectional light sheet illumination, and an Andor Neo sCMOS camera having a 2,560 × 2,160 chip of 6.5 μm pixel size. We performed serial optical imaging of the brains in a ventral-dorsal direction by exciting the FITC-albumin labeled vessels using a 488 nm diode laser and a 525/50 nm band-pass emission filter. Autofluorescence was captured with a 561 nm laser and detected via a 595/40 nm band-pass emission filter. Images of the respective brain hemisphere were acquired at 6.4× magnification with 2 μm steps in the axial direction using the dynamic focus and the highest numerical aperture of the light sheet illumination for optimal axial resolution. For image rendering of the brains, Bitplane software (Imaris, Cologne, Germany) was used. To characterize vascular changes inside the MCAO territory, regions of interest (ROIs) measuring 305 × 305 × 600 µm were selected in the ischemic and contralateral non-ischemic insular cortex +1.07 mm to +0.67 mm rostral/2.6 mm to 3.0 mm lateral/3.2 mm to 4.0 mm ventral to the bregma, which represented peri-infarct cortex in 40 min MCAO mice (Figure 1(d); see Results section). To characterize vascular changes outside the MCAO territory, we also analyzed ROIs in the S1 somatosensory (hindlimb) cortex 0.0 to −0.4 mm caudal/1.6 to 2.0 mm lateral/1.2 to 2.0 mm ventral to the bregma (Suppl. Fig. 2).

### Infarct volumetry

Brains perfused with PBS followed by PBS containing 4% PFA were frozen on dry ice and cut into 20 μm thick coronal sections. Sections were collected at 1 mm intervals for cresyl violet staining. On these sections, the border between infarcted and non-infarcted tissue was outlined using Image J (National Institutes of Health [NIH], Bethesda, MD, U.S.A.). Infarct volume was determined by subtracting the volume of the non-lesioned ipsilateral hemisphere from the volume of the contralateral hemisphere. Edema volume was calculated as volume difference between the ipsilateral and the contralateral hemisphere.

### Immunohistochemistry

20-µm-thick coronal brain sections obtained from the level of the rostral striatum (bregma +1.0 to +0.6 mm) were fixed with PBS containing 4% PFA and immersed in PBS containing 0.1% Triton X-100 and 10% normal donkey serum or 10% normal goat serum. Samples were incubated overnight at 4°C in rat anti-glycoprotein-1bα (Gp1bα; also called CD42b; M043-0; emfret, Würzburg, Germany), rabbit anti-CD31 (ab28364; abcam, Cambridge, U.K.), rabbit anti-collagen type-IV (ab756; Millipore, Burlington, MA, U.S.A.), goat anti-collagen type-IV (AB769; Sigma-Aldrich), rabbit anti-SMAα (ab5694; abcam), rabbit anti-vWF (A0082; Agilent), or rabbit anti-neuronal nuclear protein (NeuN; ab177487; abcam) antibody. Samples were rinsed and labeled with appropriate secondary Alexa Fluor-594-labeled or Alexa Fluor-488-labeled IgG, or stained with fluorescein-bound *In Situ* Cell Death Detection Kit (Roche, #11684795910) for terminal transferase dUTP nick-end labeling (TUNEL). All sections were counterstained with Hoechst 33342 (Thermo Fisher Scientific, Waltham, MA, U.S.A.). Sections were evaluated using an inverted microscope equipped with apotome optical sectioning (Axio Observer.Z1; Carl Zeiss, Oberkochen, Germany). Sections were analyzed by counting the number of TUNEL+ cells, the number of microvessel segments containing TUNEL+ cells (indicative of endothelial apoptosis), the number of collagen-IV+ microvessel segments or the number of collagen-IV+ microvessel segments containing Gp1bα+ platelet aggregates (indicative of microthrombi) in 4 ROIs of the cortex, each measuring 0.5 × 0.5 mm (Figure 1(a)).

### Vascular quantification of light sheet images

For detailed vascular quantification, stacks of 1002 images of cleared brain tissue were acquired in the ventrodorsal direction at a step size of 2 µm in the transverse plane. These stacks covered ischemic tissue of the middle cerebral artery territory and homologous non-ischemic tissue of the contralateral hemisphere. In total, a 2-mm stack of cleared brain tissue were covered by this image stack, which in non-cleared tissue represented levels 1.2 mm to 4.0 mm ventral to the bregma according to the mouse atlas by Paxinos and Franklin (http://labs.gaidi.ca/mouse-brain-atlas), taking into account brain shrinkage during the clearing procedure. In each of these image stacks, a ROI measuring 305 × 305 × 600 µm was defined in the ischemic and contralateral non-ischemic insular cortex +1.07 mm to +0.67 mm rostral to the bregma, 2.6 mm to 3.0 mm lateral to the bregma and 3.2 mm to 4.0 mm ventral to the bregma, which represented peri-infarct cortex in mice exposed to 40 min MCAO (Figure 1(d); see also ischemic injury characterization in Results section). In order to characterize vascular responses in the brain tissue outside the middle cerebral artery territory, we also analyzed a second ROI in the S1 somatosensory (hindlimb) cortex 0.0 to −0.4 mm caudal to the bregma, 1.6 to 2.0 mm lateral to the bregma and 1.2 to 2.0 mm ventral to the bregma (Suppl. Fig. 2). A major challenge of defining these ROIs was to position the 3D cubes in volumes that exhibited homogenous injury characteristics across their rostrocaudal expansion. To achieve this aim, we positioned the second ROI slightly caudal to the first one. The raw image stacks and the configuration file used for VesselExpress are available online via Zenodo (DOI: 10.5281/zenodo.10229364).

### Light sheet image preprocessing and microvascular analysis

For detailed vascular analysis image stacks were preprocessed using open source software ImageJ (National Institutes of Health, Bethesda, MD, USA) performing a rolling ball background subtraction (radius: 20 μm). In these image stacks, the microvascular labeling was analyzed. We used the image analysis pipeline VesselExpress using configurations developed to analyze brain vasculature in FITC-albumin perfused brains as described before.^12^ This pipeline makes use of a dual Frangi filter strategy for vessel segmentation, which allows analyzing diameters of small and large microvessels with high precision.^12^ For iDISCO analysis, a machine learning (ML) model with a 3D U-Net architecture^22^ was pre-trained to receive the segmentation of vessels from raw images. Vessel images and labels were pre-trained using a public dataset^12^ for 2500 epochs. Then, the vessel segmentations of raw images were predicted by the pretrained model. To generate solid vessel segmentations in hollow vessels, four images, and their corresponding vessel segmentations were chosen randomly. The vessel segmentations were filled automatically by functions ‘remove_small_objects’ and ‘closing’ from Scikit-image.^23^ Any remaining errors in the vessel segmentation were manually corrected with Napari.^24^ The pre-trained ML model was fine-tuned with the images with corrected labels for 500 epochs and then the updated model was applied to all images. To avoid any remaining small holes, a hole-filling post-processing step was applied. Using VesselExpress, a comprehensive set of network characteristics was determined, which included (a) microvascular length density (i.e., the total vessel length per brain volume), (b) branching point density (i.e., the total number of branching points per brain volume), (c) microvascular volume density (i.e., the total vessel volume per brain volume), (d) branch density (i.e. the number of branches per brain volume), (e) mean branch length between two branching points, (f) microvessel tortuosity (i.e., microvascular branch length divided by the distance between branching points), and (g) microvessel diameter. In addition, vessel length density was analyzed in small (<4 µm diameter), intermediate (4–5.4 µm diameter) and large (>5.4 µm diameter) microvessels. These caliber categories were chosen considering the distribution of diameters in the cortex. The mean diameter of microvessels was 4.7 µm. For the quantification of platelet aggregates and their association with microvessels, Imaris software (Bitplane, Oxford Instruments) was used. The Spots tool was utilized to quantify platelet aggregates with a maximum size of 9.75 µm within the ROIs. Using the Surface tool, segmented image stacks of designated small, intermediate, or large vessels were imported into Imaris. Platelet aggregates at a proximity of ≤10 µm to small, intermediate, or large microvessels were then quantified. Platelet aggregates, which did not belong to any of these three categories, were designated as platelet aggregates not associated with perfused microvessels. These platelet aggregates were likely localized in microvessels that were occluded at the moment of FITC-albumin hydrogel perfusion as a consequence of microthrombus deposits.

### Statistical analysis

Results are box plots with medians (lines)/means (plus symbols) ± interquartile ranges (IQRs) with minimum and maximum data as whiskers. Data were analyzed using two-way or one-way ANOVA followed by LSD posthoc tests. Normal distribution was confirmed by Shapiro Wilk tests (p > 0.10 for all readouts and comparisons). Equality of variances was confirmed by Levene's tests (p > 0.6 for all readouts and comparisons). *P* values ≤0.05 were considered significant. Sample sizes were calculated using a priori analysis.

## Results

### Analysis of ischemic injury

To define the degree of brain injury of MCAO mice, brain lesions were analyzed by Nissl stainings. Infarct volume in 40 min MCAO mice was 14.9 ± 5.9 mm^3^ and 22.7 ± 11.0 mm^3^ at 1 and 3 dpi, respectively. At the bregma level, these infarcts covered the striatum and lateral (parietal) cortex, which is in line with own previous studies in this stroke model.^18^ Lesion volume in 20 min MCAO mice was 4.6 ± 1.9 mm^3^ and 4.5 ± 2.3 mm^3^ at 1 and 3 dpi. These lesions were confined to the striatum in line with own earlier studies.^19^

For a more detailed analysis, we evaluated disseminate cell death by TUNEL in 4 ROIs +1.0 to +0.6 mm rostral to the bregma, which corresponds to the brain level, at which microvascular injury and remodeling were assessed by LSFM. In mice exposed to 40 min MCAO, numerous TUNEL+ (DNA-fragmented) cells were noted in the S2 somatosensory cortex at this rostrocaudal level, whereas only occasional TUNEL+ cells were found in the same ROIs in 20 min MCAO mice (Figure 1(a)). Double staining with the endothelial marker CD31 revealed a significant number of TUNEL+ microvessels in the S2 somatosensory cortex of 40 min MCAO mice, whereas few TUNEL+ microvessels were found in this area in 20 min MCAO mice (Figure 1(a)). In both MCAO models, a large number of TUNEL+ cells were found in the striatum (green fluorescence in Figure 1(a)) in line with previous studies.^18,19^

To elucidate endothelial responses to ischemia, we next stained brain sections for collagen-IV, which is expressed by endothelial cells upon ischemia/reperfusion.^25^ A significant number of collagen-IV+ microvessels were noted in the S2 and S1 somatosensory cortex, insular cortex and piriform cortex, in addition to striatum, of 40 min MCAO mice (green fluorescence in Figure 1(b)). This number was again lower in the cortex of 20 min MCAO mice, and it was confined to the striatum in most mice (Figure 1(b)). A moderate number of cortical microvessels in both MCAO models revealed Gp1bα+ platelet aggregates (red fluorescence; Figure 1(b)). This number was lower in 20 than 40 min MCAO.

To further characterize the histological responses, we outlined areas exhibiting TUNEL and collagen-IV staining (red lines in Figure 1(a) and (b)). In 40 min MCAO mice, the tissue area exhibiting collagen-IV labeling largely corresponded to the middle cerebral artery territory (including S1 and S2 somatosensory cortex dorsally and insular and piriform cortex ventrally), whereas the tissue area exhibiting DNA fragmentation was smaller and did not involve the S1 somatosensory, insular and piriform cortex (Figure 1(a) and (b)). Since the insular cortex was larger than the S1 somatosensory cortex at this rostrocaudal brain level, we defined a ROI in the insular cortex that was devoid of major cell injury, in which we performed microvascular network analyses (Figure 1(c) and (d)). Neuronal survival was analyzed in the peri-infarct cortex of mice subjected to 40 min MCAO at 1, 7 or 14 dpi. Neuronal density was not influenced by MCAO at all time-points examined (Suppl. Fig. 1).

To elucidate the effect of ischemia/reperfusion on long-term brain tissue integrity, we performed cortical volumetries on cleared brains. A moderate volume increase (by 18 ± 6% above contralateral) reflecting brain swelling was noted in 40 min but not 20 min MCAO mice at 3 dpi (Suppl. Fig. 2). Cortex volume progressively decreased until 56 dpi in 40 min MCAO mice (by 37 ± 7% compared with contralateral), whereas a more subtle decrease of cortex volume was noted in 20 min MCAO mice exhibiting predominantly striatal brain lesions (by 15 ± 23%) (Suppl. Fig. 2).

### Microvascular injury and remodeling in the peri-infarct cortex

**Figure 2.**
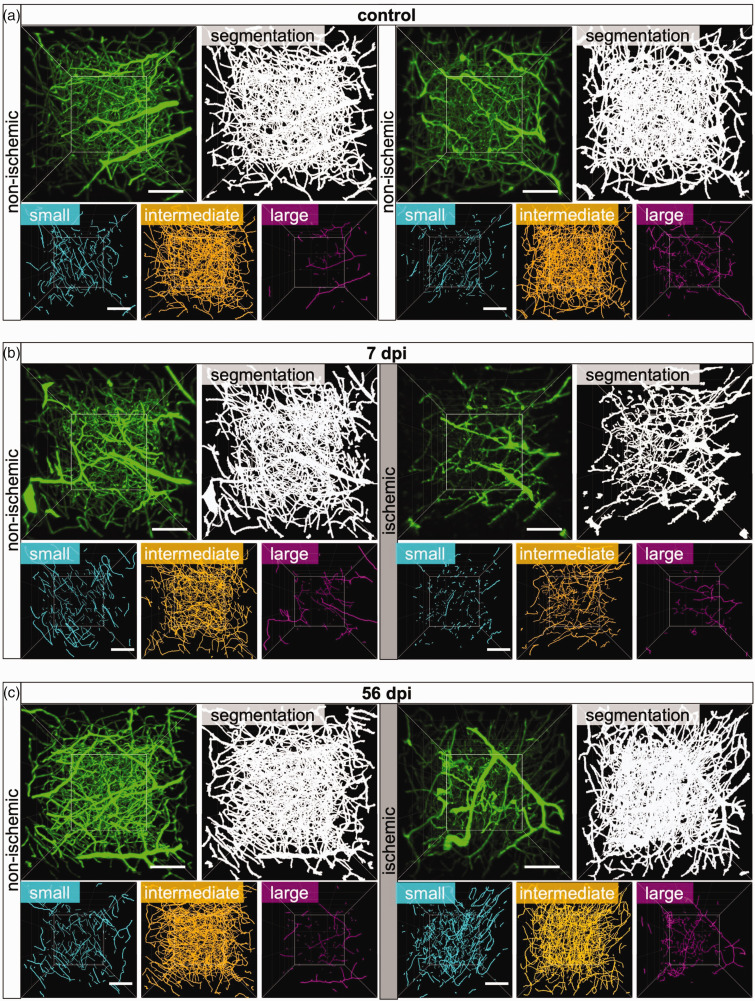
Microvascular restitution in the peri-infarct cortex is driven by small microvessels. Representative 3D stacks of original light-sheet images (green) and segmented images (white) representing a ROI in the peri-infarct cortex of (a) non-ischemic control mice or (b, c) mice exposed to 40 min MCAO followed by 7 or 56 days survival. Images for the previously ischemic peri-infarct cortex and homologous images of the contralateral non-ischemic cortex are shown. Microvessel skeletons were categorized according to vessel diameters as small (<4 µm diameter, cyan), intermediate (4–5.4 µm, orange) or large (>5.4 µm, magenta) microvessels. Note that MCAO results in the loss of small and intermediate microvessels at 7 dpi, whereas small microvessels increased above non-ischemic at 56 dpi. Scale bars: 100 µm.

We next performed microvascular analyses in this tissue area representing peri-infarct cortex in 40 min MCAO mice. Using FITC-albumin hydrogel labeling, we observed a significant reduction of vessel length density and volume density in the peri-infarct cortex at 3 and 7 dpi in 40 min MCAO mice (Figures 1(e) and 2). Of note, branching point density only modestly decreased in the peri-infarct cortex at 7 dpi (Figure 1(e)). During stroke recovery, vessel length density and volume density recovered to pre-ischemic levels within 14–56 dpi, whereas branching point density increased to values ∼2 times above pre-ischemic levels at 56 dpi (Figures 1(e) and 2). Hence, in line with our previous study on microvascular remodeling in the ischemic core,^29^ network architecture was persistently altered, reflecting a state of enhanced microvascular collateralization. The mean microvessel diameter in the non-ischemic hemisphere was very similar at all time-points (control: 4.8 ± 0.2 µm, 3 dpi: 4.7 ± 0.2 µm, 7 dpi: 4.6 ± 0.2 µm, 14 dpi: 4.7 ± 0.2 µm, 28 dpi: 4.7 ± 0.2 µm, 56 dpi: 4.7 ± 0.1 µm). In the peri-infarct cortex, a significant increase of vessel diameter was found at 7 dpi (4.9 ± 0.3 µm, p = 0.002 vs. non-ischemic) and 14 dpi (5.0 ± 0.3 µm, p = 0.030 vs. non-ischemic).

Analysis of a ROI in the more caudally located S1 hindlimb cortex outside the middle cerebral artery territory did not reveal major changes of microvascular topology in 40 min MCAO mice (Suppl. Fig. 3). Only a modest increase of vessel length density, branching point density and volume density, which failed to reach statistical significance, was noted in this area at 56 dpi.

In 20 min MCAO mice exhibiting mild ischemic injury restricted to the striatum, vessel length density and volume density, but not branching point density moderately decreased at 3 and 7 dpi (Suppl. Fig. 4). Vessel length density and volume density returned to pre-ischemic values at 14–56 dpi (Suppl. Fig. 4). Branching point density transiently increased above non-ischemic levels at 14 dpi.

### Arterial specification precedes microvascular restitution

**Figure 3.**
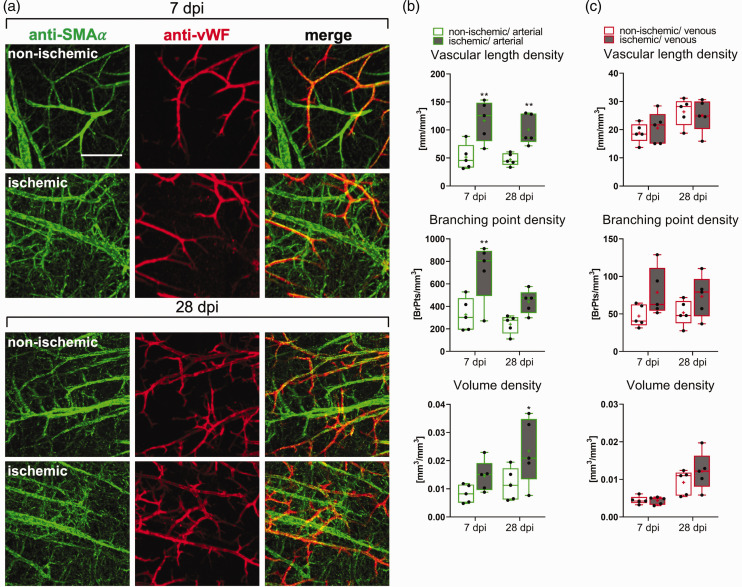
Arterial specification is an early feature of peri-infarct microvascular remodeling. (a) Maximum projections of whole brain immunolabelings for the arterial marker SMAα (green) and the venous marker vWF (red) in the previously ischemic peri-infarct cortex or contralateral non-ischemic cortex of 40 min MCAO mice. (b, c) Quantitative analysis with an enhanced VesselExpress algorithm of microvessels with arterial (SMAα+) and venous (vWF+) specification in 40 min MCAO mice. Vascular length density, branching point density, and volume density are shown. Note that the length density of microvessels with arterial specification was increased ∼2.5 times above non-ischemic levels at 7 dpi, which was the nadir of vessel length density assessed by FITC-albumin hydrogel (see Figure 1). Data are box plots with medians (lines)/means (plus symbols) ± IQRs with minimum and maximum data as whiskers. Data of individual animals are shown as dots. *p < 0.05/**p < 0.01/***p < 0.001 compared with corresponding contralateral non-ischemic. Sample size: n = 5 animals/group. Scale bar: 100 µm.

We next asked how vascular networks with arterial or venous specification were altered post-MCAO. Hence, we stained whole brains of 40 min MCAO mice sacrificed at 7 or 28 dpi with anti-SMAα and anti-VWF antibodies, which are arterial^20^ and venous^13^ specification markers, respectively. Since antibody labeling results in hollow vessel structures, which could not be evaluated using existing tools, we established an AI-based VesselExpress algorithm that is able to fill hollow vessel structures. This new algorithm now allows quantifying immunostained vessel networks (Figure 3(a)). Surprisingly, the analysis of SMAα-labeled vessels in the peri-infarct cortex revealed that the vessel length density and branching point density of microvessels with arterial specification increased by ∼2.5 times at 7 dpi (Figure 3(b)), which was the time-point of the nadir of FITC-albumin hydrogel-stained microvessels. Hence, the arterial specification of microvessels clearly preceded the reappearance of microvessels. The increased length density and branching point density of arterially specified microvessels persisted at 28 dpi (Figure 3(b)).

Of note, vWF-labeled microvessels with venous specification did not reveal any relevant changes in the peri-infarct cortex post-MCAO, except for a slight increase of branching point density at 7 and 28 dpi that did not achieve statistical significance (Figure 3(c)).

To identify if collagen-IV, which is a basal lamina constituent that provides a scaffold for cell adhesion and migration and thus aids at microvascular remodeling,^26^ was preferentially expressed on arterial or venous microvessels, brain samples of mice subjected to 40 min MCAO were doublelabeled with anti-collagen-IV and anti-SMAα or anti-collagen-IV and anti-vWF antibodies. At 1 and 7 dpi, both arterial and venous microvessels similarly exhibited collagen-IV expression in the peri-infarct cortex (Suppl. Fig. 5). Collagen-IV expression decreased thereafter and could no more be detected at 14 dpi (Suppl. Fig. 5).

### Ischemic stroke differentially affects small, intermediate and large microvessels

**Figure 4.**
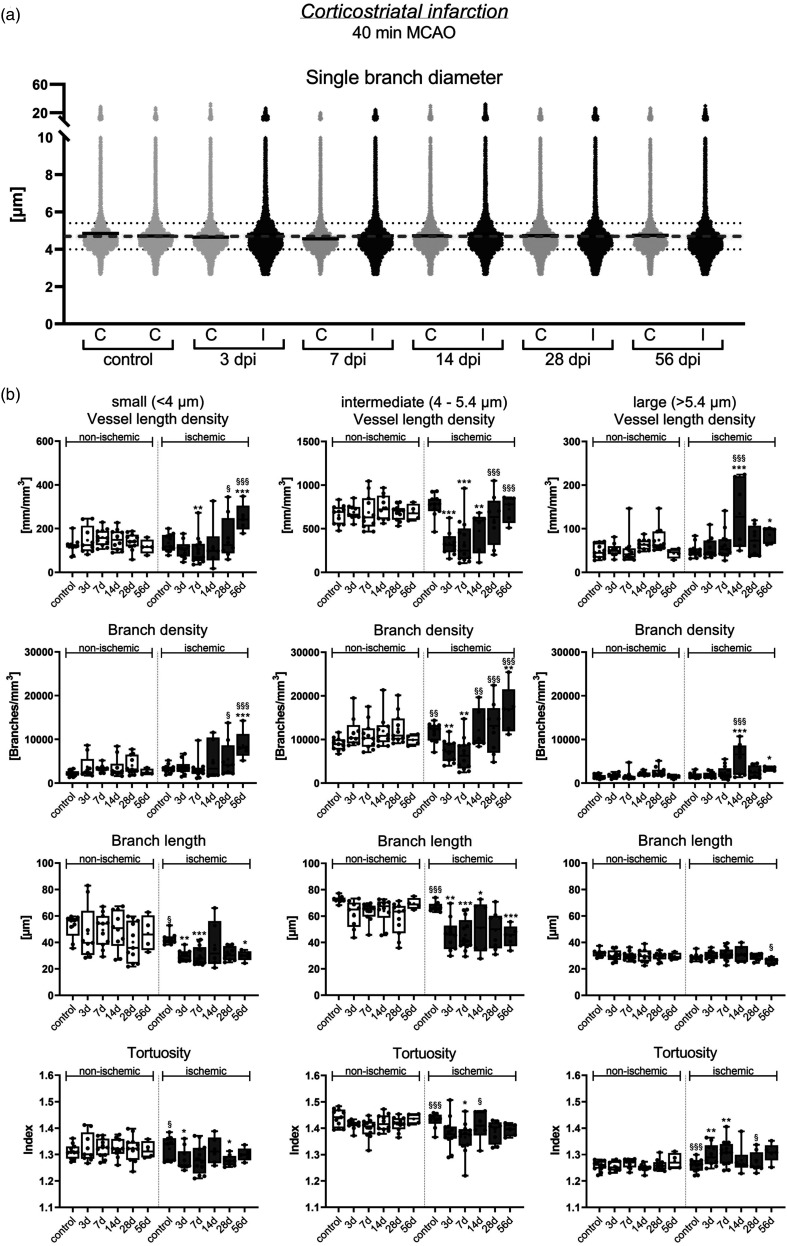
Restitution patterns of small, intermediate and large microvessels in the peri-infarct cortex. (a) Scatter dot plots of single vessel diameters showing a wide data distribution in the peri-infarct cortex and contralateral non-ischemic cortex of 40 min MCAO mice. Continuous black lines represent median values for single time-points and ROIs, dashed gray line medians of all samples (4.7 µm). Continued.Small vessels were defined as having diameters <4 µm, intermediate vessels diameters between 4 and 5.4 µm and large microvessels diameters >5.4 µm (dotted grey lines). (b) Separate analysis of small, intermediate and large microvessels in the peri-infarct cortex of 40 min MCAO mice. Note that vessel length density and mean branch length of small and intermediate microvessels decreased at 3 and 7 dpi. While the vessel length density of intermediate microvessels returned to pre-ischemic levels within 14–56 dpi, the length density of small microvessels increased ∼2 times above pre-ischemic levels. Mean branch length remained reduced across the follow-up. Data in (b) are box plots with medians (lines)/means (plus symbols) ± IQRs with minimum and maximum data as whiskers. Data of individual animals are shown as dots. *p < 0.05/**p < 0.01/***p < 0.001 compared with contralateral non-ischemic; ^§^p < 0.05/^§§^p < 0.01/ ^§§§^p < 0.001 compared with 7 dpi (i.e., nadir). Sample size: n = 11 (non-ischemic control), n = 9 (3 dpi), n = 13 (7 dpi), n = 10 (14 dpi), n = 11 (28 dpi) and n = 5 (56 dpi) animals, respectively.

We next investigated how ischemic stroke affected the network topology of small (<4.0 µm diameter), intermediate (4.0–5.4 µm diameter) and large (>5.4 µm diameter) microvessels, which represented ∼15%, ∼80% and ∼5% of the total length of all microvessels in non-ischemic brains (Figure 4(a) and (b)). The analysis of vessel characteristics in each of these size categories by FITC-albumin hydrogel revealed that the vessel length density and mean branch length of small and intermediate microvessels significantly decreased in the peri-infarct cortex of 40 min MCAO mice at 3 and 7 dpi (Figures 2 and 4(b)). The vessel length density of intermediate microvessels recovered to pre-ischemic levels from 14–56 dpi, whereas that of small microvessels increased to ∼2 times above non-ischemic levels. Mean branch length of small and intermediate microvessels was reduced across the follow-up. Branch density of intermediate microvessels was moderately reduced at 3 and 7 dpi, and branch density of small and intermediate microvessels increased above pre-ischemic levels from 14–56 dpi (Figure 4(b)), in line with the shortening of branches. The vessel length density, branch density and mean branch length of large microvessels did not change in the peri-infarct cortex at 3 or 7 dpi, but vessel length density and branch density of large microvessels moderately increased at 14 and 56 dpi, while mean branch length decreased (Figure 4(b)). Microvascular tortuosity slightly decreased in small vessels at 3, 7 and 28 dpi and increased in large vessels at 3 and 7 dpi.

The analysis of 20 min MCAO mice revealed a moderate reduction of the vessel length density of intermediate microvessels in the previously ischemic cortex at 3 and 7 dpi, which fully recovered at 14–56 dpi (not shown). The mean branch length of small and intermediate microvessels was modestly reduced at 3–56 dpi. Besides this no relevant alterations of network topology were noted.

**Figure 5.**
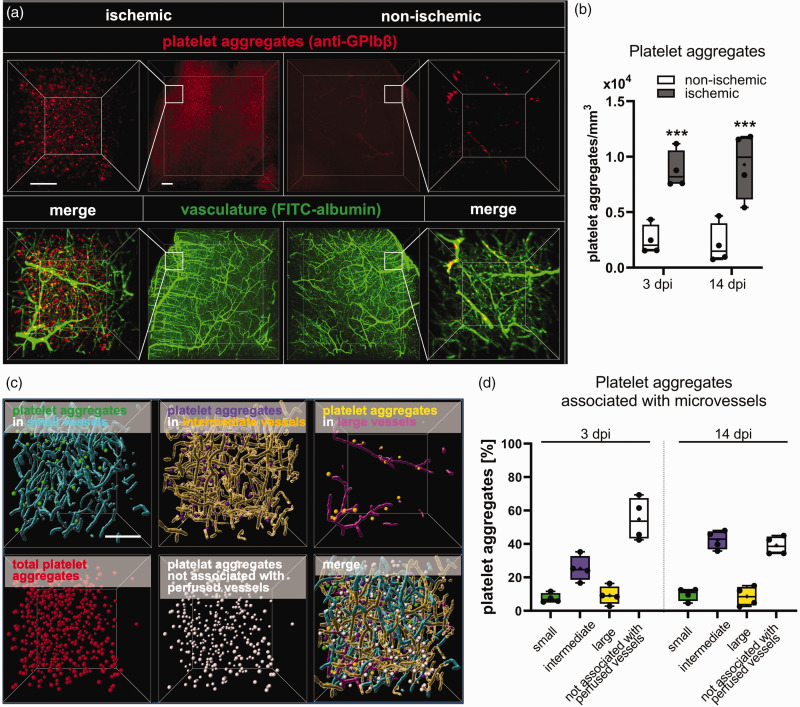
Association of platelet aggregates with small, intermediate and large microvessels. (a) Representative maximum intensity projection images showing GP1bβ-labeled platelet aggregates (in red) and FITC-albumin hydrogel-filled microvessels (in green) in the previously ischemic peri-infarct cortex (left) and the contralateral non-ischemic cortex (right). Inlets indicate analyzed ROIs. (b) Quantitative analysis in these ROIs in mice exposed to 40 min MCAO followed reveal increased platelet aggregate counts in the previously ischemic compared to contralateral non-ischemic cortex at 3 and 14 dpi. (c) Representative 3D image stacks of ROIs in the peri-infarct cortex, in which platelet aggregates were assigned to small (green spheres), intermediate (purple spheres) or large (yellow spheres) microvessels, or categorized as platelet aggregates not associated with perfused microvessels (white spheres). (d) Association of platelet aggregates with small, intermediate and large microvessels, and association with non-perfused microvessels at 3 and 14 dpi. Data in are box plots with medians (lines)/means (plus symbols) ± IQRs with minimum and maximum data as whiskers. Data of individual animals are shown as dots. ***p < 0.001 compared with contralateral non-ischemic. Sample size: n = 4 animals/group. Scale bars: 200 µm (in a, overview), 100 µm (in a, inlets, and c).

To investigate the association of platelet aggregates with small, intermediate and large microvessels, we intravitally labeled platelet aggregates by intravenous anti-GP1bβ antibody injection in 40 min MCAO mice. In cleared brains, a significant increase of platelet aggregates was detected in the peri-infarct cortex compared with non-ischemic cortex at 3 and 14 dpi (Figure 5(a) and (b)). Platelet aggregates were most frequently found in intermediate microvessels (Figure 5(c) and (d)). A significant percentage of platelet aggregates were not associated with perfused microvessels (Figure 5(c) and (d)). These platelet aggregates were likely localized in microvessels that were occluded at the moment of FITC-albumin hydrogel perfusion as a consequence of microthrombus deposits.

### FTY720 promotes microvascular restitution by increasing small microvessels

**Figure 6.**
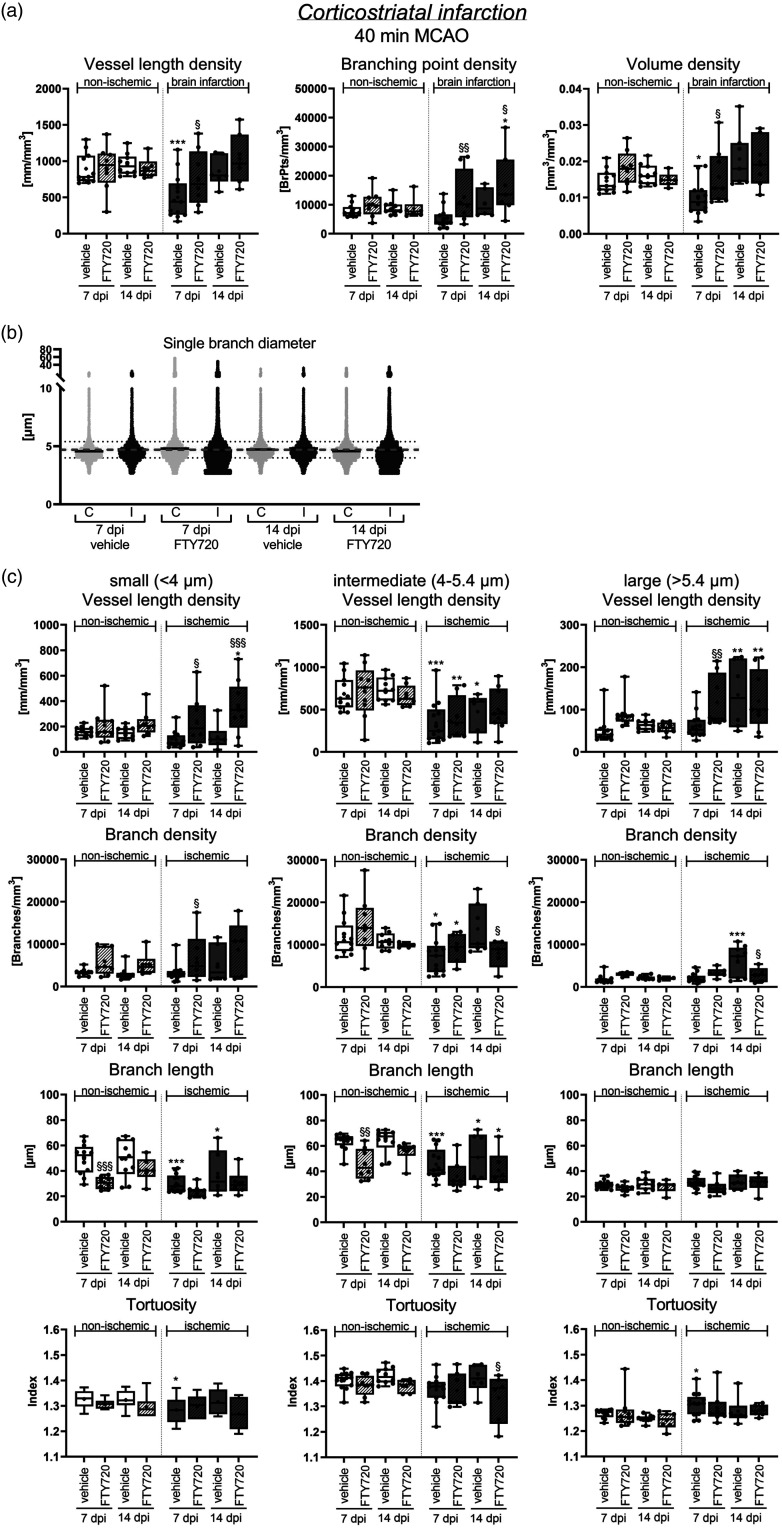
S1P analog FTY720 promotes microvascular restitution in the peri-infarct cortex by elevating small microvessels. (a) Microvascular length density, branching point density and volume density in the peri-infarct cortex and contralateral non-ischemic Continued.cortex of 40 min MCAO mice, which were i.p. treated with vehicle or FTY720 (1 mg/kg/day) starting 24 hours post-MCAO. Note that FTY720 almost completely reversed the loss of vessel length density and volume density at 7 dpi and increased branching point density above non-ischemic values at 14 dpi. (b) Scatter dot plots of single vessel diameters in the peri-infarct cortex and contralateral non-ischemic cortex of 40 min MCAO mice treated with FTY720. Continuous black lines represent median values for single time-points and ROIs, dashed gray lines medians of all samples. (c) Separate analysis of microvascular network characteristics of small, intermediate and large microvessels in the peri-infarct cortex of the same mice. Vessel length density, branch density, mean branch length and tortuosity are shown. Note that FTY720 markedly increased the vessel length density and branch density of small microvessels at 7 and 14 dpi. Data are box plots with medians (lines)/means (plus symbols) ± IQRs with minimum and maximum data as whiskers. Data of individual animals are shown as dots. *p < 0.05/**p < 0.01/***p < 0.001 compared with contralateral non-ischemic; ^§^p < 0.05/^§§^p < 0.01 compared with ischemic vehicle. Sample size: n = 12 (7 dpi, vehicle), n = 8 (7 dpi, FTY720), n = 8 (14 dpi, vehicle) and n = 6 (14 dpi, FTY720) animals, respectively.

We finally investigated how the S1P analog FTY720 influenced microvascular remodeling in the peri-infarct cortex. S1P receptors play important roles in controlling microvascular dilation and angiogenesis.^14,15^ We were therefore interested in studying FTY720’s effects on post-ischemic microvascular remodeling. Of note, FTY720 treatment almost completely reversed the loss of vessel length density and volume density and increased branching point density above levels of vehicle-treated mice in both MCAO models at 7 dpi (Figure 6(a); Suppl. Fig. 6). This effect was not attributed to post-ischemic neuroprotection. Thus, cortex volume was not influenced by FTY720 at any time-point examined (Suppl. Fig. 7). Vessel length density and volume density returned to non-ischemic levels in vehicle-treated and FTY720-treated mice at 14 dpi, whereas branching point density remained increased in 40 min MCAO mice (Figure 6(a); Suppl. Fig. 6). The separate analysis of small, intermediate and large microvessels (as above) revealed that the vessel length density of small vessels was significantly increased by FTY720 in 40 min MCAO mice at 7 and 14 dpi (Figure 6(b) and (c)). This effect was not observed in 20 min MCAO mice exhibiting striatal brain lesions (not shown).

## Discussion

This study provides an in depth characterization of microvascular network topology in the peri-infarct cortex of mice exposed to intraluminal MCAO. This was achieved using VesselExpress, an automated pipeline for the segmentation, skeletonization and graph analysis of light-sheet images.^12^ Our study shows that the post-ischemic loss of microvessels was followed by microvessel restitution over 14–56 dpi, resulting in a chronically altered network with increased branching point density. Of note, the reappearance of microvessels was preceded by the arterial specification of microvessels, as shown by SMAα immunolabeling. Evaluating vessels of different size we found that the stroke-associated loss of microvessels mainly affected small and intermediate microvessels (diameter ≤5.7 µm), while the reformation of microvessels was driven by small microvessels (<4 µm diameter). Interestingly, the clinically applicable sphingosine analog FTY720, which previously has shown beneficial effects in stroke patients,^16,17^ selectively increased the formation of small microvessels.

The loss of microvessels in the peri-infarct cortex at 3–7 dpi is supported by conventional microscopy and 2-photon microscopy studies in mice after permanent distal MCAO and photothrombotic infarcts showing microvascular occlusions in the peri-infarct tissue at 1–7 dpi.^7,27,28^ The course of microvascular recovery in our study furthermore agrees with mouse and rat conventional microscopy and 2-photon microscopy studies after MCAO and photothrombotic infarcts demonstrating microvessel restitution over 14–28 dpi.^5,7,27,28^ These earlier studies did not provide detailed insights into microvascular topology. By LSFM we found that branching point density persistently increased above pre-ischemic levels in peri-infarct microvessels, while mean branch length decreased below pre-ischemic levels. This observation corresponds to our previous LSFM observations in the ischemic core post-MCAO.^29^ The increased branching indicates a state of enhanced network collateralization, which apparently represents a signature of previously ischemic vessels.

By whole brain labeling, we for the first time demonstrated that the arterial specification of microvessels preceded the restitution of microvessels. Arterial specification defined by SMAα immunolabeling was evident in the peri-infarct cortex at 7 dpi, which was the nadir of FITC-albumin hydrogel labeling of microvessels, and it persisted at 28 dpi. SMAα is expressed on mural cells of arteries and arterioles^30^ and pericytes of arterially dedicated microvessels.^31^ Previous studies found that SMAα is increased in peri-infarct tissue of transient proximal MCAO rats from 1–28 dpi.^32–34^ Its association with angiogenesis was not shown. SMAα is involved in blood flow regulation via vasocontraction or vasodilation.^30^ It may be hypothesized that SMAα-associated vascular tone responses set the stage for microvessel formation, which is in line with earlier studies demonstrating a role of pericytes in post-ischemic brain tissue recovery.^35^

Our observation that small to intermediate microvessels are predominantly lost in peri-infarct tissue is in line with conventional immunohistochemistry^36^ and own LSFM^10^ studies. In earlier LSFM studies, we segmented microvessels used the Imaris tool,^10^ which overestimates microvessel diameters.^12^ In that study we found that vessels with a diameter of <10 µm were reduced in the reperfused ischemic brain tissue.^10^ Using the dual Frangi filter segmentation strategy employed by VesselExpress, which allows evaluating small microvessels with high accuracy,^12^ we now narrowed down this diameter to <5.7 µm. An important finding of our study is that the restitution of microvessels post-MCAO was driven by the reappearance of small microvessels (<4.0 µm diameter), which increased ∼2 times above non-ischemic levels. Of note, the S1P analog FTY720 selectively increased the density of small microvessels. Previous studies revealed that FTY720 increased microvascular patency,^37,38^ blood-brain barrier integrity,^37,38^ endothelial proliferation^39^ and microvascular density^29^ post-MCAO. In our study, FTY720 treatment was initiated 24 hours post-MCAO. Hence, the promotion of microvascular remodeling was not attributed to acute cerebroprotection. It is likely that endothelial S1P receptors mediated FTY720’s angiogenic action.^14,15^

Our study provides unprecedented details of microvascular remodeling in the peri-infarct cortex, which likely influence long-term tissue outcome. Thus, persistent network changes were noted in MCAO mice indicative of an increased network collateralization, which were augmented by the S1P analog FTY720. We did not examine implications of microvascular remodeling for cerebral hemodynamics and functional neurological recovery. Additional studies are warranted on these issues. LSFM significantly advances our understanding of restorative processes post-MCAO and will greatly facilitate the evaluation of new stroke therapies.

## Supplemental Material

sj-pdf-1-jcb-10.1177_0271678X241270407 - Supplemental material for Arterial specification precedes microvascular restitution in the peri-infarct cortex that is driven by small microvessels Supplemental material, sj-pdf-1-jcb-10.1177_0271678X241270407 for Arterial specification precedes microvascular restitution in the peri-infarct cortex that is driven by small microvessels by Nina Hagemann, Yachao Qi, Ayan Mohamud Yusuf, AnRan Li, Xiaoni Zhang, Philippa Spangenberg, Anthony Squire, Thorsten R Doeppner, Fengyan Jin, Shuo Zhao, Jianxu Chen, Axel Mosig, Matthias Gunzer and Dirk M Hermann in Journal of Cerebral Blood Flow & Metabolism
