# Supplementary material for: Arterial specification precedes microvascular restitution in the peri-infarct cortex that is driven by small microvessels
Source: J Cereb Blood Flow Metab. 2024 Aug 7;45(1):171–86. doi: 10.1177/0271678X241270407 (PMC11571960; doi:10.1177/0271678X241270407)
Supplement: sj-pdf-1-jcb-10.1177_0271678X241270407 - Supplemental material for Arterial specification precedes microvascular restitution in the peri-infarct cortex that is driven by small microvessels [file sj-pdf-1-jcb-10.1177_0271678X241270407.pdf]

Arterial specification precedes microvascular restitution in the periinfarct cortex that is driven by small microvessels

Nina Hagemann, PhD[1,2], Yachao Qi, MD[1,2], Ayan Mohamud Yusuf, PhD[1,2], AnRan Li; MSc[1,2], Xiaoni Zhang, MSc[1,2], Philippa Spangenberg, MSc[3,4], Anthony Squire, PhD[3,4], Thorsten R. Doeppner, MD[1,2], Fengyan Jin, MD[5], Shuo Zhao; MSc[6], Jianxu Chen; PhD[6], Axel Mosig, PhD[7], Matthias Gunzer, PhD[3,4,6], Dirk M. Hermann, MD[1,2]*

[1]Department of Neurology, University Hospital Essen, Essen, Germany [2]Center for Translational Neuro- and Behavioral Sciences, University Hospital Essen, Essen, Germany [3]Institute for Experimental Immunology and Imaging, University Hospital Essen, Essen, Germany, [4]Imaging Center Essen, University Hospital Essen, Essen, Germany; [5]Department of Hematology, First Hospital of Jilin University, Changchun, China; [6]Leibniz-Institut für Analytische Wissenschaften (ISAS), Dortmund, Germany, [7]Bioinformatics Group, Center for Protein Diagnostics, Ruhr-University, Bochum, Germany

*Correspondence: Dirk M. Hermann, MD, Department of Neurology, University Hospital Essen, Hufelandstr. 55, DE-45147 Essen, Germany, dirk.hermann@uk-essen.de

Supplemental Figures

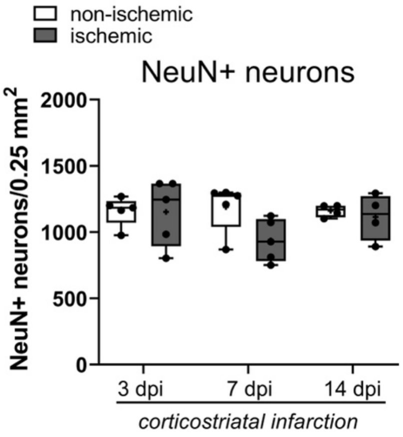

Supplemental Fig. 1 Neuronal density analysis in the peri-infarct cortex. Density of NeuN+ neurons in the peri-infarct cortex of 40 min MCAO mice at 3, 7 and 14 days post-ischemia (dpi). Data are box plots with medians (lines)/means (plus symbols) ± IQRs with minimum and maximum data as whiskers. Data of individual animals are shown as dots. Sample size: n=5 (3 dpi), n=4 (7 dpi) and n=4 (14 dpi) animals, respectively.

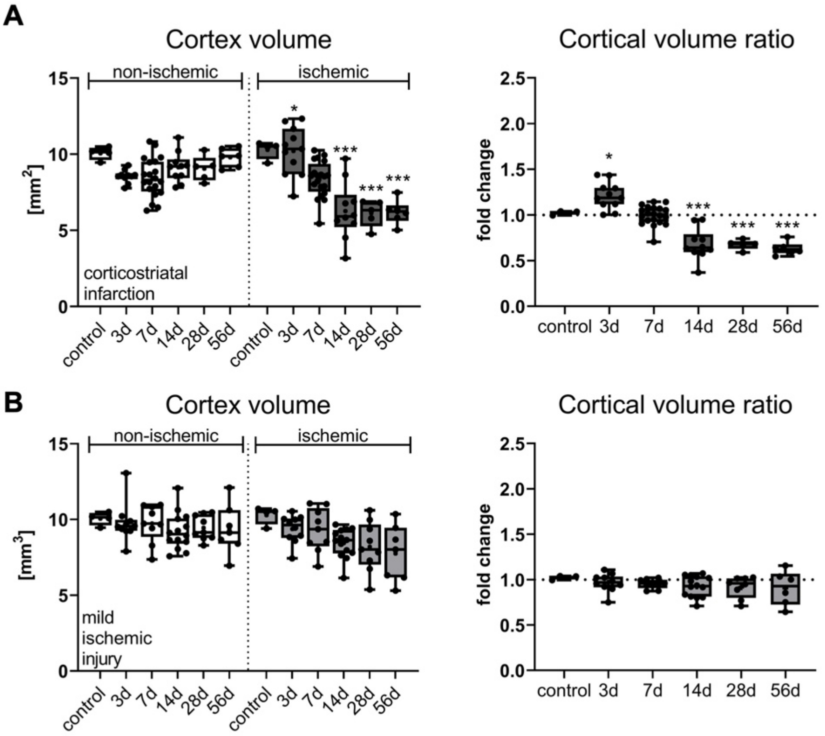

Supplemental Fig. 2 Cortical volumetry of mice exhibiting corticostriatal infarction and striatal brain injury. Volume analysis of the previously ischemic and contralateral non-ischemic cortex (left) as well as volume ratio of the ischemic vs. contralateral non-ischemic cortex (right) of mice subjected to **(A)** 40 min or **(B)** 20 min middle cerebral artery occlusion (MCAO). The cortical volume was outlined using the 561 nm autofluorescence signal. Note the moderate volume increase (indicating cortex swelling) at 3 dpi that was followed by more profound volume shrinkage (reflecting cortex atrophy) from 14 to 56 dpi in 40 min MCAO mice exhibiting corticostriatal infarction, which is contrasted with the slight volume reduction in 20 min MCAO mice exhibiting mild ischemic striatal brain injury. Data are box plots with medians (lines)/ means (plus symbols) ± IQRs with minimum and maximum data as whiskers. Data of individual animals are shown as dots. *$p<0.05$/***$p<0.001$ compared with contralateral non-ischemic (left) or with non-ischemic control (right). Sample size in (**A**): n=4 (non-ischemic control), n=10 (3 dpi), n=20 (7 dpi), n=10 (14 dpi), n=5 (28 dpi) and n=6 (56 dpi) animals, respectively; sample size in (**B**): n=4 (non-ischemic control), n=10 (3 dpi), n=9 (7 dpi), n=14 (14 dpi), n=9 (28 dpi) and n=7 (56 dpi) animals, respectively.

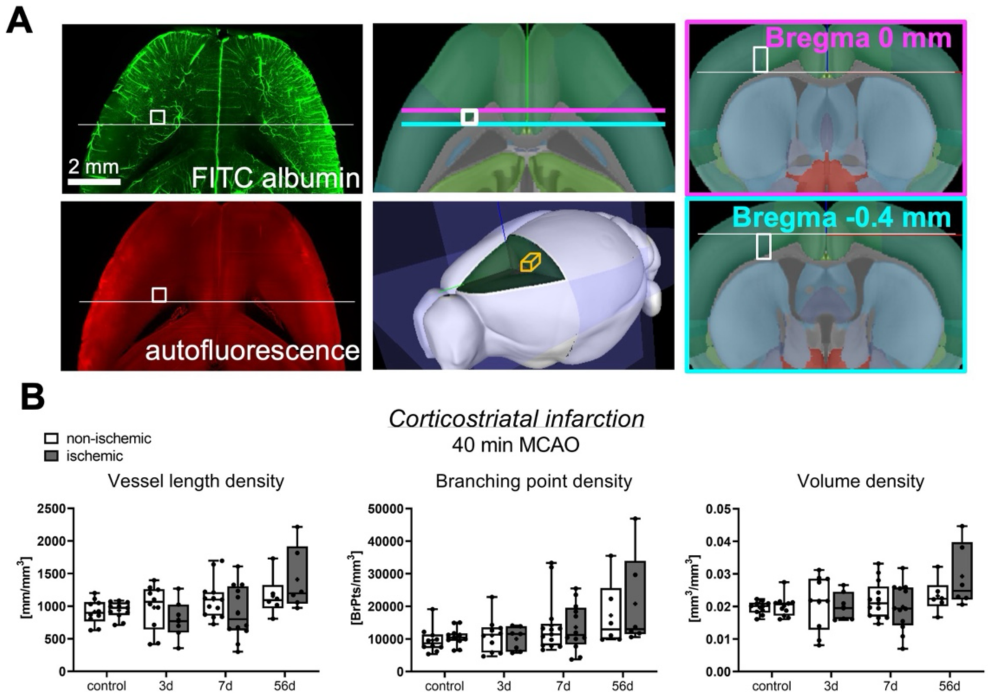

Supplemental Fig. 3 Microvascular network characteristics in the S1 hindlimb cortex outside the middle cerebral artery territory of mice exhibiting corticostriatal infarction. (A) Left: Representative images of brains in transverse (scanning) direction showing FITC-albumin stained vessels (top) or 561 nm autofluorescence (bottom). Center: Allen Mouse Brain Atlas images representing transverse view (top) or whole brain view displaying location of region of interest (ROI) in the S1 hindlimb cortex used for vascular analysis. Right: Coronal view displaying the ROI at its starting point and endpoint. According to the immunohistochemical analyses shown in Figure 1, this ROI was located outside the middle cerebral artery territory. Scale bar, 2 mm. **(B)** Microvascular network analysis in the non-ischemic S1 somatosensory cortex outside the middle cerebral artery territory of 40 min MCAO mice assessed by FITC-albumin hydrogel. Microvascular length density, branching point density and volume density are shown at 3, 7 or 56 dpi. Data are box plots with medians (lines)/ means (plus symbols) ± IQRs with minimum and maximum data as whiskers. Data of individual animals are shown as dots. No significant group differences were detected. Sample size: n=10 (non-ischemic control), n=10 (3 dpi), n=13 (7 dpi) and n=6 (56 dpi) animals, respectively.

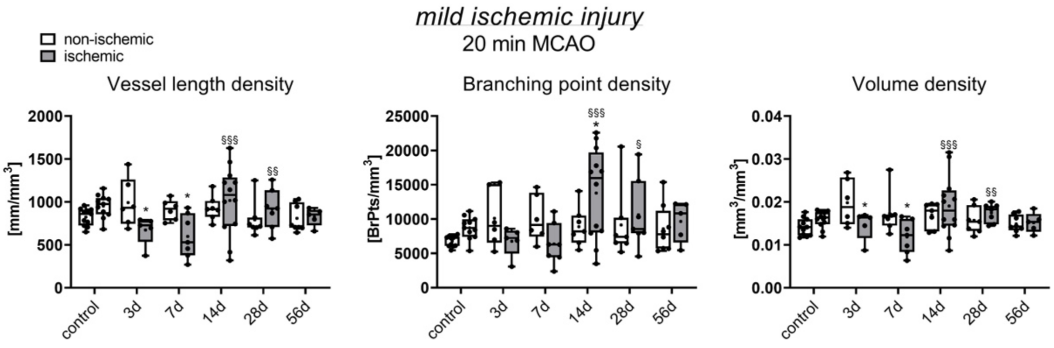

Supplemental Fig. 4 Microvascular network characteristics in previously ischemic cortex of mice exhibiting striatal brain injury. Microvascular network characteristics in the previously ischemic and contralateral non-ischemic cortex of mice exposed to 20 min MCAO. Microvascular length density, branching point density and volume density were assessed by FITC-albumin hydrogel at 3, 7, 14, 28 or 56 dpi. Note the moderate reduction of the vessel length density and volume density at 3 and 7 dpi, which fully recovered at 14 to 56 dpi. Note the transient elevation of branching point density at 14 dpi. Data are box plots with medians (lines)/ means (plus symbols) ± IQRs with minimum and maximum data as whiskers. Data of individual animals are shown as dots. *p<0.05/**p<0.01/***p<0.001 compared with contralateral non-ischemic; §p<0.05/§§p<0.01/§§§p<0.001 compared with 7 dpi (i.e., nadir). Sample size: n=11 (non-ischemic control), n=6 (3 dpi), n=7 (7 dpi), n=8 (14 dpi), n=7 (28 dpi) and n=5 (56 dpi) animals, respectively.

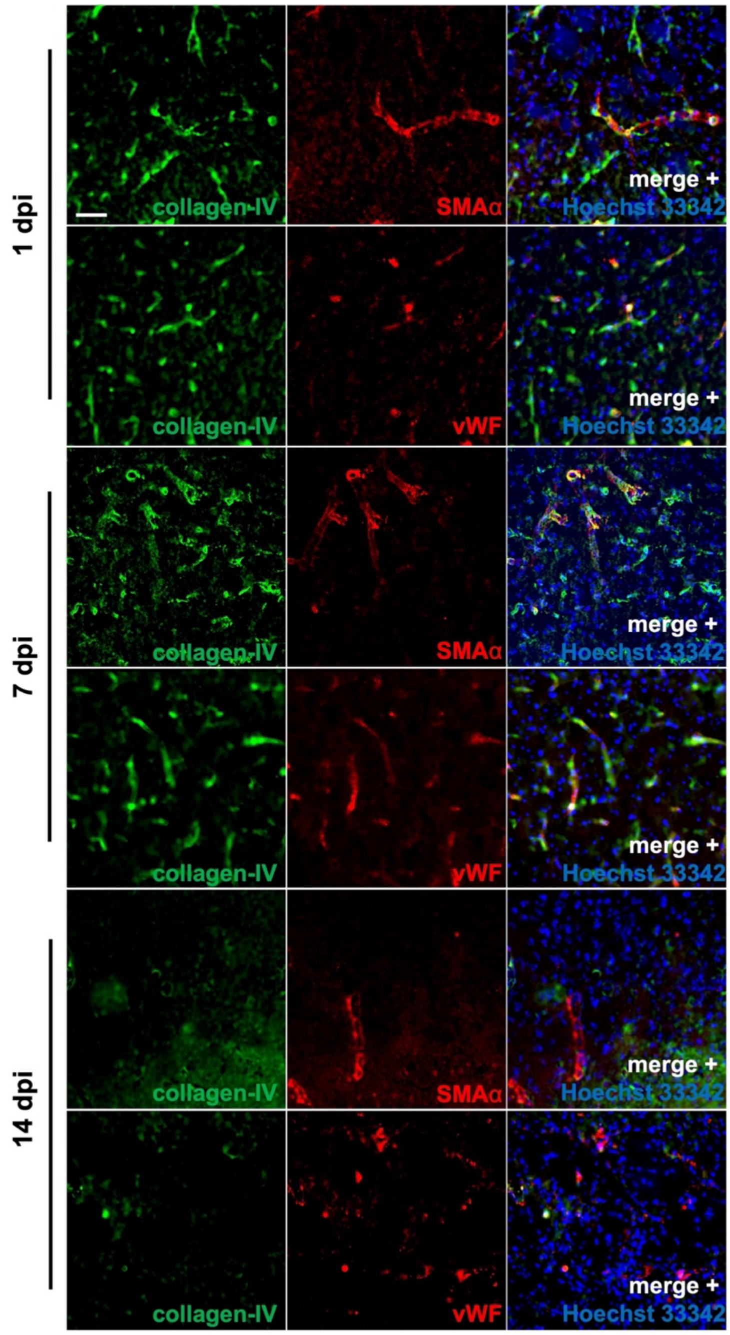

Supplemental Fig. 5 Expression of collagen-IV on arterially and venously specified ischemic microvessels. Immunolabeling of the basal lamina protein collagen-IV, the arterial specification marker smooth muscle actin (SMAα) and the venous specification marker von Willebrand Factor (vWF) in the brains of mice exposed to 40 min MCAO. Representative images show colocalization of collagen-IV with arterial (SMAα+) and venous (vWF+) microvessels at 1 and 7 dpi. Scale bar: 50 µm.

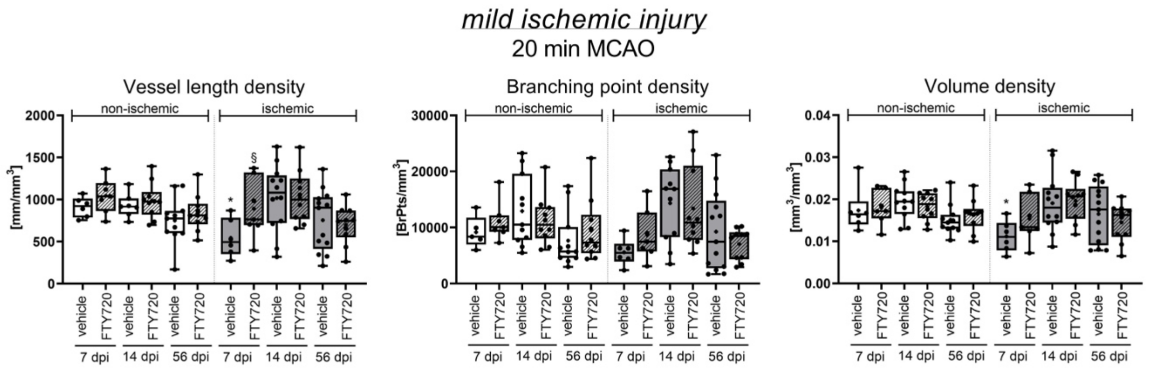

Supplemental Fig. 6 Effects of S1P analog FTY720 on microvascular remodeling in the previously ischemic cortex of mice exhibiting striatal brain injury. Microvascular length density, branching point density and volume density in the previously-ischemic cortex and contralateral non-ischemic cortex of mice exposed to 20 min MCAO, which were i.p. treated with vehicle or FTY720 (1 mg/kg/day) starting 24 hours post-MCAO. Note that FTY720 increased vessel length density and volume density at 7, but not 14 dpi. Data are box plots with medians (lines)/ means (plus symbols) ± IQRs with minimum and maximum data as whiskers. Data of individual animals are shown as dots. *$p<0.05$ compared with contralateral non-ischemic; §$p<0.05$ compared with ischemic vehicle. Sample size: n=6 (7 dpi, vehicle), n=7 (7 dpi, FTY720), n=7 (14 dpi, vehicle), n=10 (14 dpi, FTY720), n=11 (56 dpi, vehicle) and n=10 (56 dpi, FTY720) animals, respectively.

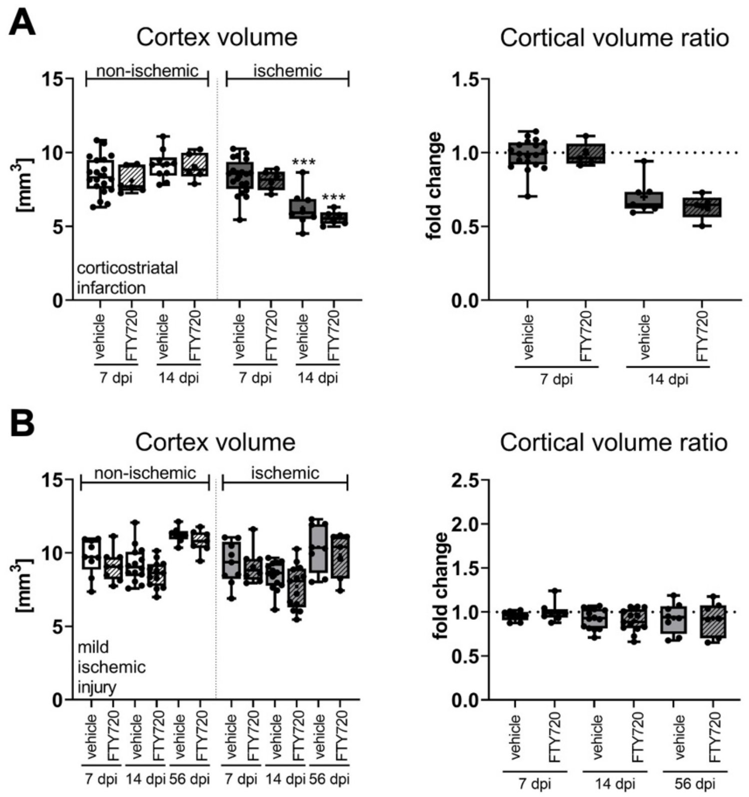

Supplemental Fig. 7 Cortical volumetry of ischemic mice treated with FTY720. Volume analysis of the previously ischemic and contralateral non-ischemic cortex (left) as well as volume ratio of the ischemic vs. contralateral cortex of mice subjected to **(A)** 40 min or **(B)** 20 min MCAO, which were i.p. treated with vehicle or FTY720 (1 mg/kg/day) starting 24 hours post-MCAO. Note that FTY720 did not influence cortex volume or shrinkage at any time-point examined. Data are box plots with medians (lines)/ means (plus symbols) ± IQRs with minimum and maximum data as whiskers. Data of individual animals are shown as dots. No significant differences between vehicle-treated and FTY720-treated mice were detected. Sample size: n=9 (7 dpi, vehicle), n=8 (7 dpi, FTY720), n=14 (14 dpi, vehicle), n=13 (14 dpi, FTY720), n=8 (56 dpi, vehicle) and n=7 (56 dpi, FTY720) animals, respectively.

Supplemental Table 1: Animal numbers and exclusion reasons

Study	Animal cohort	Experimental intervention	Animal sex	Animal age	Animal number (prior to experiment)	Animal number (end of experiment)	Reason for animal exclusion
LSFM	Cohort 1	Non-ischemic control	Male	9-12 weeks	11	11	
	Cohort 2	brain infarction, 3 dpi	Male	9-12 weeks	12	12	
	Cohort 3	brain infarction, 7 dpi	Male	9-12 weeks	20	15	4 animals prematurely sacrificed due to predefined endpoint criteria defining excessive animal suffering, 1 brain could not be cleared for LSFM
	Cohort 4	brain infarction, 14 dpi	Male	9-12 weeks	15	10	5 animals prematurely sacrificed due to predefined endpoint criteria
	Cohort 5	brain infarction, 28 dpi	Male	9-12 weeks	15	11	2 animals prematurely sacrificed due to predefined endpoint criteria, 2 animals could not successfully be perfused with FITC-albumin
	Cohort 6	brain infarction, 56 dpi	Male	9-12 weeks	8	5	3 animals prematurely sacrificed due to predefined endpoint criteria
	Cohort 7	brain infarction, 7 dpi, vehicle	Male	9-12 weeks	12	12	
	Cohort 8	brain infarction, 7 dpi, FTY720	Male	9-12 weeks	10	8	2 animals prematurely sacrificed due to predefined endpoint criteria
	Cohort 9	brain infarction, 14 dpi, vehicle	Male	9-12 weeks	10	8	2 animals prematurely sacrificed due to predefined endpoint criteria

	Cohort 10	brain infarction, 14 dpi, FTY720	Male	9-12 weeks	10	6	4 animals prematurely sacrificed due to predefined endpoint criteria
	Cohort 11	brain infarction, 3 dpi, anti-GP1b injection	Male	9-12 weeks	5	4	1 animal prematurely sacrificed due to predefined endpoint criteria
	Cohort 12	brain infarction, 14 dpi, anti-GP1b injection	Male	9-12 weeks	5	4	1 animal prematurely sacrificed due to predefined endpoint criteria
	Cohort 13	mild ischemic injury, 3 dpi	Male	9-12 weeks	11	10	1 animal prematurely sacrificed due to predefined endpoint criteria
	Cohort 14	mild ischemic injury, 7 dpi	Male	9-12 weeks	21	20	1 animal prematurely sacrificed due to predefined endpoint criteria
	Cohort 15	mild ischemic injury, 14 dpi	Male	9-12 weeks	11	10	1 animal could not successfully be perfused with FITC-albumin
	Cohort 16	mild ischemic injury, 28 dpi	Male	9-12 weeks	10	5	3 animals prematurely sacrificed due to predefined endpoint criteria, 2 brains could not successfully be cleared for LSFM
	Cohort 17	mild ischemic injury, 56 dpi	Male	9-12 weeks	10	6	2 animals prematurely sacrificed due to predefined endpoint criteria, 2 animals could not successfully be perfused with FITC-albumin
	Cohort 18	mild ischemic injury, 7 dpi, vehicle	Male	9-12 weeks	10	6	1 animal prematurely sacrificed due to predefined endpoint criteria, 3 brains could not be cleared
	Cohort 19	mild ischemic injury, 7 dpi, FTY720	Male	9-12 weeks	10	7	2 animals prematurely sacrificed due to predefined endpoint criteria, 1 animal could not successfully

							be perfused with FITC-albumin
	Cohort 20	mild ischemic injury, 14 dpi, vehicle	Male	9-12 weeks	10	7	1 animal prematurely sacrificed due to predefined endpoint criteria, 2 animals could not successfully be perfused with FITC-albumin
	Cohort 21	mild ischemic injury, 14 dpi, FTY720	Male	9-12 weeks	10	10	
	Cohort 22	mild ischemic injury, 56 dpi, vehicle	Male	9-12 weeks	15	11	3 animals prematurely sacrificed due to predefined endpoint criteria, 1 brain could not be cleared
	Cohort 23	mild ischemic injury, 56 dpi, FTY720	Male	9-12 weeks	15	10	2 animals prematurely sacrificed due to predefined endpoint criteria, 3 animals could not successfully be perfused with FITC-albumin
Immunohistochemistry	Cohort 24	brain infarction, 1 dpi	Male	9-12 weeks	8	7	1 animal prematurely sacrificed due to predefined endpoint criteria
	Cohort 25	brain infarction, 3 dpi	Male	9-12 weeks	8	8	
	Cohort 26	brain infarction, 7 dpi	Male	9-12 weeks	8	8	
	Cohort 27	brain infarction, 14 dpi	Male	9-12 weeks	8	8	
	Cohort 28	mild ischemic injury, 1 dpi	Male	9-12 weeks	8	7	1 animal prematurely sacrificed due to predefined endpoint criteria
	Cohort 29	mild ischemic injury, 3 dpi	Male	9-12 weeks	8	6	2 animals prematurely sacrificed due to predefined endpoint criteria

iDISCO	Cohort 30	brain infarction, 7 dpi	Male	9-12 weeks	7	5	1 animal prematurely sacrificed due to predefined endpoint criteria, 1 animal could not successfully be perfused with FITC-albumin
	Cohort 31	brain infarction, 28 dpi	Male	9-12 weeks	7	5	1 animal prematurely sacrificed due to predefined endpoint criteria, 1 animal could not successfully be perfused with FITC-albumin
